# Development of a practical aggregate spatial road freight modal demand model system for truck and commodity movements with an application of a distance-based charging regime

**DOI:** 10.1007/s11116-022-10271-2

**Published:** 2022-03-16

**Authors:** David A. Hensher, Edward Wei, Wen Liu, Loan Ho, Chinh Ho

**Affiliations:** Institute of Transport and Logistics Studies (ITLS), The University of Sydney Business School, Sydney, NSW 2006 Australia

**Keywords:** Freight models, Commodity distribution, Aggregate truck type shares, Australia, Distance based-charging

## Abstract

We promote a view that more attention should be given to the freight sector in order to recognise that many initiatives designed to impact on passenger travel do also impact on the performance of the movement of freight vehicles and hence the ability to distribute commodities from the locations in which they are generated. This paper develops a practical freight demand model system and applies the models within an integrated passenger and freight model system for the Greater Sydney Metropolitan Area using a distance based charge for trucks and cars as a way of highlighting the importance of not ignoring truck traffic when assessing road pricing reform in the car passenger sector.

## Introduction

The distribution of freight is a crucial feature of modern society. Between 2007 and 2016, the Australian domestic freight task grew by 50%, with 726 billion tonne kilometres of freight moved in 2015–2016 representing an annual average of 30,000 tonne kilometres for every person in Australia (Bureau of Infrastructure Transport and Regional Economics 2018). Close to 34% of all freight tonne kilometres, bulk plus non-bulk, in Australia are carried by trucks with trucks dominating in the non-bulk sector. The freight task is forecast to grow by an additional 26% between 2016 and 2026,^1^ adding to congestion in the road network, especially approaching and within metropolitan areas.

The current interest in freight distribution by road stems from a plan to find a practical way of incorporating the freight task into an integrated transport and land use model system, be it with a metropolitan or regional/rural focus, which historically, with rare exception, has focussed on passenger travel activity. We promote a view that attention should be given to the freight sector within strategic transport model systems in order to recognise that many initiatives designed to impact on passenger travel do also impact on the performance of the movement of freight vehicles, and hence the ability to distribute commodities from the locations from which they are generated. De Jong et al. (2021) has recently reviewed the broader literature on this theme, reinforcing the integration objective of this paper. The focus of this paper is in showing how an analyst can develop a practical policy sensitive freight model system that can be integrated into a strategic transport model system together with passenger models using available aggregate data sources.

In studying freight distribution activity, we need to identify the volume of freight being moved by commodity class between the spatially defined origins and destinations of a study area, and the amount and share of truck movements by type of road vehicle (namely rigid and articulated trucks) associated with each commodity class. Allowance for empty trucks is also of great relevance. While this may seem like a straightforward specification of the essential elements of a practical freight movement demand model system, the identification of appropriate data in many countries is quite a challenge. In the Australian context, there are a number of candidate data sources, but no one source provides all the data required. For example, while at least two data sources provide volumes of commodities transported between an agreed spatial representation of origins and destinations, the commodity classes are not strictly comparable; and one data set does not split kilo-tonnes by rigid and articulated trucks, and only one data set separates demand volume by port and domestic movement, the latter important to account for the import and export setting of freight movement.

In this paper, we share the journey of data sourcing, data incompatibility and ways in which data can be merged in order to take advantage of the strengths of specific data sets. All this data preparation is guided by the models that have to be estimated in a format that makes them spatially and output-wise compatible with two integrated transport and land use model systems into which they will be positioned; a metropolitan focussed system called Metroscan, developed at The University of Sydney (Ho et al. 2017; Hensher et al. 2020a, b, also Appendix A in the current paper), and a national and Statewide model system called RTresis^2^ that incorporates metropolitan, rural and regional contexts. Importantly, the metropolitan model must recognise that some freight flows begin or end in origins or destinations that are outside of the metropolitan area and hence external travel zones must be included to capture these features of freight movements. MetroScan and RTresis feed demand forecasts into a benefit–cost and economic analysis framework as shown schematically in Appendix A and more detail in Hensher et al. (2020a).

An important interaction between truck movements and passenger activity is through the volumes of vehicle movements on the road network. Within the traffic assignment procedure in MetroScan, we have to load all passenger and freight truck movements onto the road network, and together with the service levels on each link, obtain equilibrium levels of traffic movement and the associated performance metrics such as travel times and delays associated with congestion. Accounting for freight movement,,^3^ is of great relevance and failure to do so results in an inability in benefit–cost and economic impact analyses to capture the fuller productivity benefits from improved transport infrastructure investment and other policy initiatives, a major source of wider economic impact.

The US *Quick Response Freight Methods* (QRFM) approach (Beagan et al. 2019) emphasises freight usage and performance, and the need to integrate freight demand models into a broader integrated transport and land use planning and policy system like MetroScan or the Oregon State Transportation and Land Use Model Integration Program (TLUMIP) (Donnelly et al. 2018), or the number of systems developed in Europe (summarised in de Jong et al. 2021), all of which recognise that other vehicles share networks with freight such as light commercial and passenger vehicles. Freight truck travel times reflect the congestion times in many localities, resulting from those other demands. As Beagan et al. (2019, page 10) state “Unless planners include the usage and performance of these other components of travel, it may not be possible to forecast freight demand and performance.”

This paper and its contribution is organised as follows. We begin by setting out the model framework within which to capture the key components of freight and truck movement.^4^ This is followed by a detailed presentation of the data items required and where they were sourced from, and what assumptions were required to merge the data sources in order to have a data set compliant with the modelling requirements. A descriptive profile of the data is then provided, followed by the estimated models and the interpretation of the main findings. To ensure that the models are able to be built into MetroScan, they have to be calibrated to the base year vehicle type shares and overall frequency of movements by commodity class. We explain how this is done, and the final models are presented ready for implementation. A case study is for the Greater Sydney Metropolitan area is set out, with the freight model system integrated into MetroScan with an application of a distance-based road user charge with separate rates per kilometre applied to trucks and passenger cars.

Importantly, we emphasise that there are many sophisticated freight demand model systems developed by researchers; however many, but not all, of these have faced challenges in implementing within an integrated (passenger and freight) transport and land use model system, often due to the available data. The essential elements are represented in our model system. Some general references on this topics are Cambridge Systematics (1997), de Jong et al. (2004, 2016, 2021), Expedite Consortium (2000), Holguín-Veras and Patil (2008), Nuzzolo (2013), Tavasszy (1994), Tavasszy and De Jong (2013), Wisetjindawat (2006), Bovy and Jansen (1983), Ozbay et al. (2005), Gonzalez-Calderon et.al. (2017), Gonzalez-Calderon et al. (2021), Holguín-Veras et al. (2021), Hunt and Stefan (2007), Nowreen et al. (2018), and Takanori et al. (2020).

## The Road Freight Movement Model Framework

**Fig. 1:**
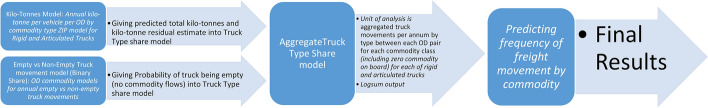
The structure of the model system and linkages

Four models are proposed as the representation of the main behavioural features of truck and commodity movements between spatially defined locations (Fig. 1). We need to know the total numbers of vehicles by class moving between each origin and destination, and the amount of each commodity within a defined set of classes that is carried by each vehicle type. Truck movements often do not carry freight^5^ and hence we have to allow for empty truck movements. This will enable us to predict the amount of freight vehicle movement activity as well as volumes of commodities being transported under a reference setting and a scenario setting, where the latter enables changes in freight distribution due in particular to exogenous socio-demographic influences such as population and employment forecasted changes, and changes in the transport network (e.g., improvements in the road network). A diagrammatic overview of the linkages between these models is given in Fig. 1 and also in the Appendix A schematic diagram for the interface with the overall Metroscan model system (Hensher et al. 2020a, b) where the freight component is embedded with passenger and land use models.

Formally, the quantity, *X*, of total truck movements for each of type *k* commodities transported, including empty truck movements (and hence zero kilo-tonnes transported), from origin zone *i* to destination *j* by truck class *m,* $X_{ijm}^{k}$, can be expressed as Eq. (1).

1 $$X_{ijm}^{k} = X_{ij}^{k} \frac{{exp\left( {U_{ijm}^{k} } \right)}}{{\mathop \sum \nolimits_{m} exp\left( {U_{ijm}^{k} } \right)}}$$

$X_{ij}^{k}$ is the quantity of overall truck movements associated with commodities of type *k* produced in origin zone *i* and transported to destination zone *j* (by the distribution model), as well as zero commodity movement in empty trucks, and $U_{ijm}^{k}$ is the observed utility of transporting commodity *k* from origin zone *i* to destination zone *j* by truck class *m*.

### Aggregate truck type share model

The representative utility associated with the aggregate truck type share specification in (1) can be decomposed into the additive observed and unobserved influences of relative utility as in Eq. (2).

2 $$U_{ijm}^{k} = V_{ijm}^{k} +\epsilon_{ijm}^{k}$$

${\text{V}}_{ijm}^{k}$ represents the observed influences such as the volume of freight moved (in kilo-tonnes), the incidence of empty trucks, and level of service variables such as travel time between each *ij* pair, as well socio-economic influences such as population size that are specific to location *i* or *j*; and $\epsilon_{ijm}^{k}$ is an index of the aggregate unobserved influences, assumed to be represented in the sampled population as an extreme value type 1 distribution which, under the independent and identically distributed (IID) assumption, results in the popular multinomial logit (MNL) model form. Importantly, to ensure full clarity of the form in which the MNL model is implemented, given the aggregate nature of the frequency data, we do not refer to a ‘truck type choice’ model, but to an ‘aggregate truck share’ model. In Nlogit6, the software used (Greene 2016, Chapter N-321), frequency data consists of a set of frequency counts for the outcomes. Such frequencies are non-negative integers for the outcomes in the choice set and may be zero.

### Aggregate truck movement model

$X_{ij}^{k}$ in Eq. (1) represents the annual frequency of aggregate truck movements for each commodity class, whose functional form is now discussed. Movement frequency is a positive number compliant with a count model such as zero inflation Poisson (ZIP)^6^ and heterogeneity. Using standard regression methods, as is common in truck generation models, is not strictly correct. Truck movements are strictly count variables and should not be considered as continuous variables. We do, however, understand that some authors assimilate them as such in large scale applications with large counts. Estimation of a Poisson model instead of a linear regression is a very straightforward approach. Not only does it avoid an irrelevant estimation method based on a somewhat weak law of large numbers when it is not an empirical case, it is a correct scientific statistical approach.

The frequency model is connected to the aggregate truck type share model by the expected maximum utility (also referred to as logsum and defined as ln*$\mathop \sum \limits_{m} exp\left( {U_{ijm}^{k} } \right) {\text{Hensher et al}}. 2015$) associated with the aggregate truck type share model. The total frequency of truck movements is a non-negative count value. For a discrete random variable, *Y,* observed over a period of length *T*_*n*_ and observed frequencies, *y*_*n*_, (*n* observations), and explanatory variables **x**_*n*_ (e.g., logsum), the Poisson regression model shown as Eq. (3) as given in Greene (2000).

3 $$\Pr ob(Y = \mathop y\nolimits_{n} |\mathop x\nolimits_{n} ) = \frac{{\exp ( - \mathop \lambda \nolimits_{n} )\mathop {\mathop \lambda \nolimits_{n} }\nolimits_{{}}^{{\mathop y\nolimits_{n} }} }}{{\mathop y\nolimits_{n}^{^{\prime}} !}},\mathop y\nolimits_{n} = 0,1,...;\log \mathop \lambda \nolimits_{n} = \mathop \beta \nolimits^{^{\prime}} \mathop x\nolimits_{n}$$

λ_*n*_ is both the mean and variance of *y*_*n*_; *E*[*y*_*n*_|**x**_*n*_] = λ_*n*_. We allow for unobserved heterogeneity to recognise the possibility of partial observability if data on observed trip activity at the commodity and OD level exhibits zero trips. Specifically, the answer ‘zero’ could arise from two underlying responses. If we were unable to capture any truck movements between a specific origin–destination (OD) pair within a commodity class, we would only observe a zero; however, the zero may be due to the measurement period and the response might be some positive number in other periods. We define *z* = 0 if the response would always be 0, 1 if a Poisson model applies; *y* = the response from the Poisson model; then *zy* = the observed response. The probabilities of the various outcomes in the ZIP model are shown in (4).

4a $$\Pr ob[y = 0] = \Pr ob[z = 0] + \Pr ob[z = 1]*\Pr ob[y = 0|Poisson]$$

4b $$\Pr ob[y = r > 0] = \Pr ob[z = 1]*\Pr ob[y = r|Poisson]$$

The ZIP model is given as (Greene 2012) *Y*_*n*_ = 0 with probability *q*_*n*_; *Y*_*i*_ ~ Poisson (λ_n_) with probability 1 – *q*_*n*_ so that Prob[*Y*_*n*_ = 0] = *q*_*n*_ + [1 – *q*_*n*_]*R*_*n*_(0) and Prob[*Y*_*n*_ = *r* > 0] = [1 – *q*_*n*_]*R*_*n*_(*r*). *R*_*n*_(*y*) = the Poisson probability = e ^−λ*n*^ λ_*n*_^*yn*^/y_*n*_! and λ_*n*_ = e^**β′x***n*^. We assume that the ancillary, state probability, *q*_*n*_, is distributed logistically; *q*_*n*_ ~ Logistic [*v*_*n*_]. Let *F*[*v*_*n*_] denote the logistic CDF. Then, *ʋ*_*i*_ can be defined by the form in Eq. (5) labelled the ZIP(τ) model (Greene 2012).

5 $$v_{n} \; = \;\tau {\text{log}}[\lambda_{n} ]\,{ = }\,\tau \beta^{\prime}{\mathbf{x}}_{n}$$

τ may be positive or negative and if there is evidence of zero trips in any observations, the τ parameter is expected to be statistically significant; otherwise the Poisson form with normal latent heterogeneity is adopted.

### The kilo-tonnes model

The annual kilo-tonnes per OD pair and commodity class^7^ model is designed to recognise that exogenous changes, such as the growth in population and employment, will change the volume of commodities by class that need to be distributed. A zero inflated Poisson regression model is estimated of the same form as presented above for truck movement frequency. Any change in kilo-tonnes due to an exogenous shock will influence the vehicle type shares, and through the logsum obtained from the aggregate truck type share model, will impact on aggregate truck movements between each OD pair for a commodity class. There remains the possibility that kilo-tonnes is an endogenous influence on the aggregate truck share, as in the presence of empty trucks, and so we have to test for this possibility. There are a number of ways to set up a discrete choice share model that embeds the presence of endogeneity associated with a specific inclusion in the representative component of a utility expression (see Train and Wilson (2009), Guevara et al. (2019), and Wooldridge (2015)).

Control functions are statistical methods to correct for endogeneity by modelling the endogeneity in the relevant random components (Wooldridge 2015; Hensher et al. 2021). This involves two stages: (i) the endogenous variable is regressed on exogenous instruments; then, (ii) the residual (or a function of it) is incorporated into the utility function of the aggregate truck type share model as an explanatory variable denoted the control. An advantage of the control function approach is that the test that the parameter on the control function is zero is equivalent to a test of exogeneity. For the Poisson regression model, the generalized residual is r_qi_ = Y(i) − Exp(beta'x(i)) = Observed(i) − Expected(i).

### Aggregate truck movement frequency

To predict changes in aggregate truck movement frequency, the logsum (or expected maximum utility) associated with each OD pair and commodity class as an output from the aggregate truck type share model can be used as a predictor variable in a frequency (truck movement) model for each commodity class, as a way of predicting the overall movement frequency.

The logsum from the aggregate truck type share model provides the mechanism for linking two models in a nested structure that aligns with an underlying theory of random utility maximisation (see Hensher et al. 2015). Specifically, our model system enables us to link the truck movement shares with the absolute frequency of all truck movements between each OD pair for a given commodity class. The movement frequency is a positive number which is compliant with a count model. We estimated the frequency count model with the logsum as a predictor.

### Model estimation order

The order of model estimation is as follows (see Fig. 1). *Firstly,* the kilo-tonne models are estimated for each commodity class and truck type at the OD pair level. Given that we want to establish whether kilo-tonnes in the aggregate truck type share model is endogenous, the residuals from the Poisson regressions, which are now control functions, are saved and are included together with the kilo-tonne variable in the truck type model.

*Secondly*, a separate model is estimated to distinguish empty and commodity-laden aggregate truck movements at the OD pair level. If the residuals are statistically significant, this suggests that there are correlated errors between the random errors of the aggregate truck type share models and the errors of the kilo-tonne model, which are now purged through the inclusion of these residuals.^8^ If the residuals are not statistically significant, then the exogeneity assumption is assumed to hold.

*Thirdly,* a multinomial logit model using frequency counts for each truck type is estimated. The dependent variable is aggregated truck movements per annum by type between each OD pair for each commodity class (including zero commodity on board) for each of rigid and articulated trucks. The previous two models are linked using the predicted kilo-tonnes and the probability of empty truck movements. Residuals are tested for the presence or otherwise of endogeneity associated with the prior two models.

*Finally*, the logsum obtained from the aggregate truck type share model becomes an explanatory variable in the aggregate truck movement Poisson regression model. We now move to discuss the data sources used to construct the data set used in model estimation.

## Sourcing and constructing a road freight database for Australia

Developing the aggregated spatial freight modal demand model system requires that we have several key elements in the dataset. *First*, the dataset should cover freight movement regions within Australia with details provided for both origins and destinations such as population and other socio-demographics for characterising the location that may have an impact on freight movement. *Second*, the dataset should also apply a widely adopted list of commodity types with which freights movements, kilo tonnes of goods, kilometres travelled and other freight information can be classified. *Third*, the dataset should allow filtering information by alternative modes, in this instance, rigid and articulated trucks. With the dataset containing the above three types of data, it can be used to model the relationship between frequency of movement and amount of freight by commodity type, truck type, and other information to inform how commodities are moved from one location to another.

No single available data source can provide all the required information. We were able to source and combine three data sets to construct the required freight dataset: (1) the road freight data set from the Australian Bureau of Statistics (ABS, 2014); (2) the “Strategic Freight Movement” (SFM) data set from Transport Performance and Analytics (TPA, 2016) of Transport for New South Wales; and (3) the number of movements by vehicle for both rigid and articulated trucks from TPA (TPA, 2016). The key characteristics of the three data sets are summarised in Table 1.^9^

**Table 1:** The summary of the three data sources for annual data

ABS	SFM	TPA
Year of the survey 2014	Year of forecast 2016	Year of forecast 2016
Mode (road)	Mode (road, rail)	Mode (road, rail)
Origin	Origin	Origin
Destination	Destination	Destination
Commodity (23 items)	Commodity (49 items)	Commodity (49 items)
Vehicle group (rigid, articulated trucks)		Vehicle group (rigid, articulated trucks)
Total km travelled	Total kilo tonnes	Number of movements (tMove) between OD each commodity each vehicle
Total tonnes carried		
Total km tonnes		

The ABS data set contains road freight data obtained from a survey conducted in 2014. The data include total kilometres travelled, total tonnes carried, and total kilometre tonnes for both rigid and articulated trucks for 23 commodity items, across 351 SA3^10^ level origins and destinations (see maps in Appendix B) for an entire year. It does not contain truck movement frequency between each OD pair by either commodity type or truck type.

The SFM data set contains forecasted freight demand, at the SA3 level, for the 40-year period between 2016 and 2056 for road and other modes. We extracted 2016 data from this data sets to have a close match with the other two data sets. The data includes annual kilo tonnes for each OD pair for a total of 49 commodities.^11^ This commodity list is different from the one used by ABS but can be reclassified to the ABS types. This dataset does not provide separate freight kilo tonnes for each truck type, providing only the total kilo tonnes for each commodity class. The TPA data set provides the movement frequency by vehicle type which was missing in the ABS and SFM databases. The remaining fields in the data set correspond to the SFM database matching by origin, destination and commodity type.

## Descriptive profile of the combined data

A descriptive profile of the data ready for model estimation, as a merging of the three data sets, is summarised in Tables 2 and 3. It is the most complete data set currently available in Australia for estimating the aggregate spatial models presented in a previous section.

**Table 2:** Summary of annual number of rigid and articulated truck movements for each commodity class (across OD pairs)

	Rigid	Articulated	Total	Rigid %	Artic %
Empty truck movements	3,408,972	4,143,627	7,552,599	45.14	54.86
Machinery and transport equipment	2,190,369	829,149	3,019,518	72.54	27.46
Food (animal or human consumption)	1,480,632	1,516,203	2,996,835	49.41	50.59
Cork and wood	1,397,555	1,085,588	2,483,143	56.28	43.72
Crude materials	1,469,137	743,308	2,212,445	66.40	33.60
Sand, stone and gravel	610,563	1,021,400	1,631,963	37.41	62.59
Petroleum and petroleum products	753,421	561,586	1,315,007	57.29	42.71
Cement and concrete	396,061	895,020	1,291,081	30.68	69.32
Coal	0	1,265,620	1,265,620	0.00	100.00
Chemicals	873,438	330,848	1,204,286	72.53	27.47
Miscellaneous manufactured articles	457,054	493,465	950,519	48.08	51.92
Tools of trade	347,038	487,290	834,328	41.59	58.41
Cereal grains	308,657	490,878	799,535	38.60	61.40
General freight	125,545	602,517	728,062	17.24	82.76
Beverages and tobacco	279,975	121,196	401,171	69.79	30.21
Live animals	208,582	122,519	331,101	63.00	37.00
Metalliferous ores and metal scrap	115,444	140,431	255,875	45.12	54.88
Iron and steel	30,377	84,976	115,353	26.33	73.67
Other manufactured articles	81,525	18,415	99,940	81.57	18.43
Animal and vegetable oils, fats and waxes	2,964	24,651	27,615	10.73	89.27
Other commodity	201,452	805,746	1,007,198	20.00	80.00
Total	14,738,761	15,784,433	30,523,194	48.29	51.71

**Table 3:** Summary statistics of annual kilo tonnes per vehicle per OD pair by commodity type

	Rigid			Articulated		
Commodities	Mean	SD	CV	Mean	SD	CV
Cereal grains	71.54	160.29	224.06	58.3	137.64	236.09
Food (animal or human consumption)	935	25.59	273.69	9.66	36.5	377.85
Live animals	3.74	11.04	295.19	2.54	7.91	311.42
Beverages and tobacco	2.8	7.81	278.93	3.16	10.24	324.05
Crude materials	6.32	16.43	259.97	7.37	17.7	240.16
Metalliferous ores and metal scrap	4.43	20.22	456.43	11.79	90.75	769.72
Sand, stone and gravel	13.84	28.6	206.65	14.91	28.25	189.47
Cock and wood	5.26	26.87	510.84	7.44	37.32	501.61
Tools of trade	6.38	24.87	389.81	7.47	26.34	352.61
Petroleum and petroleum products	13.17	31.03	235.61	14.11	31.92	226.22
Animal and vegetable oils. fats and waxes	9.43	11.4	120.89	4.15	6.99	168.43
Chemicals	4.27	11.44	267.92	4.77	11.93	250.10
Cement and concrete	5.52	11.93	216.12	11.18	17.75	158.77
Iron and steel	3.89	8.87	228.02	5.21	7.6	145.87
Other manufactured articles	1.39	2.37	170.50	1.41	2.44	173.05
Machinery and transport equipment	6.57	22.3	339.42	6.51	19.08	293.09
Miscellaneous manufactured articles	13.45	34.84	259.03	20.86	42.4	203.26
General freight	50.33	57.61	114.46	63.9	83.41	130.53
Other commodity	30.89	114.87	371.87	31.97	118.97	372.13
Total	7.79	29.63	380.36	9.89	65.4	661.27

With the exception of coal, all commodities are transported by a mix of rigid and articulated trucks, with food, followed closely by cork and wood, and crude materials, contributing the most to the truck movement task, with the exception of empty trucks. There is a good overall share of truck movements between the two classes of vehicles for all commodities, except coal, with rigid trucks dominating, in particular, for other manufactured articles, chemicals, machinery and transport equipment. Excluding coal, general freight and animal and vegetable oils, fats and waxes are mainly moved by articulated vehicles. 24.74% of all truck movements do not carry any commodities,^12^ indicating vividly the opportunity to use this spare capacity through improved scheduling and greater cooperation amongst trucking companies. Two categories, “natural and manufactured gases” and “manufactured fertilisers” did not record movements by rigid and articulated trucks.

The volume of commodities carried per vehicle in kilo-tonnes per annum per vehicle per OD pair is shown in Table 3, noting that the product of the Table 3 OD volumes and Table 2 truck movements can be used to obtain the annual total volume of commodities transported in each commodity class and vehicle type. The standard deviation numbers relative to the mean are of particular interest since they show the variation in volumes carried per truck type, with the greatest variation (defined by the coefficient of variation (CV), which is the Standard Deviation/Mean*100) being for cork and wood for rigid vehicles and cereal and grains for articulated trucks. There is a significant amount of variability in the volumes carried by each vehicle class within each commodity class. The profiles in Table 2 and 3 describe the dependent variables used in the models to be estimated that we now turn to.

## Model results

### The zero-inflated poisson model for annual kilo-tonnes per vehicle per OD pair

The first model in the system describes annual kilo-tonnes per vehicle between each OD pair for rigid and articulated trucks using a Zero-Inflated Poisson (ZIP) model, given the distribution of the dependent variable. There is a highly skewed distribution of kilo-tonnes between each OD pair for each commodity class; being towards zero (empty truck movements^13^) and covering rigid and articulated trucks combined, with a mean of 8.05 per vehicle, a standard deviation of 51.32 and 1, 1, 5, and 17 respectively for 25, 50, 75 and 90 percentiles. As the dependent variable, kilo-tonnes can neither be modelled using ordinary least squares regression or an unaltered Poisson model, which would demand either a normal or a Poisson distribution. The resulting ZIP models for both rigid and articulated trucks are shown in Table 4. The Vuong statistics of 26.13 and 24.87 suggest that the ZIP model is strongly favoured over an unaltered Poisson model for both truck types.

**Table 4:** Model results for the annual kilo-tonnes per vehicle per OD by commodity type (ZIP model) for Rigid and Articulated Trucks

Variables	Acronyms	Units	Parameter estimates		t-value		Parameter estimates	t-value
Poisson regression model			Rigid truck				Articulated truck	
Constant			2.7659		389.72		2.5981	448.41
Population of work people at origin	OWORK	in '000	0.0002		95.45		0.0006	694.83
Population 2016 at destination	DPOP16T	in '000	0.0004		225.09		0.0004	290.63
Destination in New South Wales	DNSW	Dummy (1/0)	0.4007		58.08		0.3724	67.59
Food (animal or human consumption)	FOOD	Dummy (1/0)	− 0.9427		− 276.15		− 0.9055	− 385.55
Live animals	LIVEANIM	Dummy (1/0)	− 1.6222		− 196.68		− 1.9286	− 266.18
Beverages and tobacco	BEVTOB	Dummy (1/0)	− 1.7929		− 208.69		− 1.8911	− 264.10
Crude materials	CRUDEMAT	Dummy (1/0)	− 1.3508		− 306.86		− 1.3184	− 409.49
Metalliferous ores and metal scrap	METORES	Dummy (1/0)	− 1.9205		− 90.35		− 1.8833	− 101.19
Sand, stone and gravel	SAND	Dummy (1/0)	− 0.6972		− 150.32		− 0.3826	− 132.26
Cork and wood	CORKWOOD	Dummy (1/0)	− 1.7570		− 359.25		− 1.7106	− 457.84
Tools of trade	TTRADE	Dummy (1/0)	− 1.3459		− 281.88		− 1.5194	− 370.69
Petroleum and petroleum products	PETROL	Dummy (1/0)	− 0.2289		− 53.55		− 0.1960	− 61.53
Chemicals	CHEMICAL	Dummy (1/0)	− 1.6938		− 305.03		− 1.7452	− 357.48
Cement and concrete	CEMCONCR	Dummy (1/0)	− 1.4136		− 244.53		− 0.6101	− 194.03
Iron and steel	IRONSTEL	Dummy (1/0)	− 1.8203		− 99.95		− 1.3792	− 79.49
Other manufactured articles	OTHEMANU	Dummy (1/0)	− 2.4425		− 84.4		− 2.3153	− 90.57
Machinery and transport equipment	MACHTRPT	Dummy (1/0)	− 1.4937		− 346.82		− 1.6436	− 481.23
Miscellaneous manufactured articles	MISC	Dummy (1/0)	− 0.4854		− 111.08		− 0.3823	− 128.95
Other commodities	OTHCOM3	Dummy (1/0)	− 0.7013		− 138.24		− 0.8986	− 165.06
Zero inflation Model								
Tau			− 0.2706		− 31.13		− 0.7063	− 82.53
Model fits								
Log− likelihood for ZIP			− 88,321.50			1,358,99.83		
Log-likelihood for Poisson			− 1,622,99.83			− 256,164.48		
Sample size			13,394			17,081		
Vuong statistics (ZIP vs. unaltered model)			26.13			24.87		

Model test: Vuong statistics > 1.96 favours the ZIP model over the unaltered Poisson model

The volume of kilo-tonnes per vehicle per annum transported between each OD pair is statistically influenced by the population at the destination, which is a good indicator of the demand for commodities, with a larger population demanding more kilo-tonnes of commodities. In addition, we have a statistically significant effect that is positive for the kilo-tonnes distributed to locations in New South Wales compared to the other States. We do not have a measure of jobs at each origin, but as a proxy we use the number of people in the population who work, and although they may not work in the same zone in which they live, we assume that the statistical significance of the variable is a proxy for the catchment area in which individuals work. We know that there is a very high correlation between where workers live and where the jobs are even if it is not the same individuals in both definitions.

## Accounting for empty truck movements

As shown in Table 2, there are many truck trips where no commodities are being moved. Although empty truck movements are treated as zero volumes of freight between each OD pair inTable 4,^14^ we need to develop an additional model to identify the incidence of the number of empty trucks as influenced by the volumes of commodities, as well as the production and attraction factors such as population as a good proxy from economic activity and location specific effects. The probability of empty truck trips can be modelled as a function of commodity flows of various commodities, and other influences, following the suggestions in the literature (e.g., Holguín-Veras & Thorson 2003; Holguín-Veras & Patil 2008).

Table 5 presents the two aggregate logit share models, given the aggregate frequency of truck movements for each truck type, in predicting the probability of a truck being empty versus carrying commodities, between each OD pair, with one model for the rigid truck and the other model for the articulated truck. The model findings show that the flows of some commodity classes are statistically significant in predicting the probability of trucks being empty versus non-empty between each OD pair. For example, the positive parameter estimate for live animal flows suggests that the more movement of live animals occurs, the higher is the probability that some trips between specific OD pairs for rigid and articulated trucks will move empty (often associated with the backhaul). Conversely, where petroleum and petroleum products are being transported, a higher volume of distribution tends to reduce the probability of empty rigid and articulated trucks. These findings highlight the importance of accounting for the commodity movement profile at the OD level in conditioning the likelihood of having vehicles on the road that are not transporting any product.

**Table 5:** Aggregate logit OD commodity models for annual empty vs non-empty truck movements

Variables	Acronyms	Units	Parameter Estimates	t-value	Parameter Estimates	t-value
			Rigid Truck		Articulated Truck	
Constant	Constant		− 3.0872	− 24.41	− 2.3581	− 26.15
Cereal grains	KTCERG	kilo-tonnes per OD per annum	− 0.0005	− 0.20	− 0.0013	− 0.44
Food (animal or human consumption)	KTFOOD	kilo-tonnes per OD per annum	− 0.0084	− 1.81	0.0032	1.00
Live animals	KTLIVEAN	kilo-tonnes per OD per annum	0.1483	4.92	0.1250	4.87
Beverages and tobacco	KTBEVTO	kilo-tonnes per OD per annum	0.0602	2.66	0.0207	1.31
Crude materials	KTCRUDE	kilo-tonnes per OD per annum	− 0.0004	− 0.06	0.0180	2.02
Metalliferous ores & metal scrap	KTMETOR	kilo-tonnes per OD per annum	− 0.0115	− 1.20	− 0.0002	− 0.05
Sand, stone and gravel	KTSAND	kilo-tonnes per OD per annum	0.0030	0.60	0.0120	4.11
Cork and wood	KTCORK	kilo-tonnes per OD per annum	0.0133	2.56	0.0044	2.18
Tools of trade	KTTRADE	kilo-tonnes per OD per annum	0.0040	0.97	0.0013	0.35
Petroleum and petroleum products	KTPETROL	kilo-tonnes per OD per annum	− 0.0136	− 2.55	− 0.0078	− 2.33
Animal and vegetable oils, fats and waxes	KTANVE	kilo-tonnes per OD per annum	0.1162	2.47	0.0867	2.02
Chemicals	KTCHEM	kilo-tonnes per OD per annum	− 0.0372	− 2.53	− 0.0415	− 3.06
Cement and concrete	KTCEMCON	kilo-tonnes per OD per annum	0.0134	1.66	0.0054	1.33
Iron and steel	KTIRON	kilo-tonnes per OD per annum	− 0.2389	− 1.80	− 0.2552	− 2.64
Other manufactured articles	KTOTHM	kilo-tonnes per OD per annum	− 0.0717	− 0.61	0.0069	0.08
Machinery and transport equipment	KTMACHTR	kilo-tonnes per OD per annum	0.0011	0.25	− 0.0082	− 1.82
Miscellaneous manufactured articles	KTMISC	kilo-tonnes per OD per annum	0.0008	0.23	0.0038	1.52
General freight	KTGENFR	kilo-tonnes per OD per annum	0.0159	1.18	0.0174	0.97
Other commodity	KTOTHER	kilo-tonnes per OD per annum	0.0034	1.09	0.0079	1.27
NSW as origin	ONSW	Dummy (1/0)	0.1586	2.58	− 0.3572	− 3.60
NSW as destination	DNSW	Dummy (1/0)	0.2088	3.40	0.6769	6.82
VIC as origin	OVIC	Dummy (1/0)	0.0775	0.41	− 0.0925	− 0.82
VIC as destination	DVIC	Dummy (1/0)	0.5143	2.67	1.0351	9.04
QLD as origin	OQLD	Dummy (1/0)	0.0344	0.18	0.0201	0.18
QLD as destination	DQLD	Dummy (1/0)	0.4641	2.46	0.7767	6.75
SA as origin	OSA	Dummy (1/0)	0.2869	1.42	− 0.1623	− 1.35
SA as destination	DSA	Dummy (1/0)	0.2447	1.23	0.8860	7.32
WA as origin	OWA	Dummy (1/0)	0.5756	0.80	0.0859	0.40
WA as destination	DWA	Dummy (1/0)	− 0.0325	− 0.05	0.8659	4.02
Population for origin	OPOP16M	million people	− 2.8051	− 3.62	− 3.2673	− 6.13
Population for destination	DPOP16M	million people	− 1.4082	− 2.80	− 3.6818	− 6.27
**Model fits**						
Log likelihood			− 33,93.69		− 5,242.59	
McFadden Pseudo-R^2^			0.274		0.254	
Sample size			7,103		10,779	
AIC/N			0.97		0.98	

We also find that there are some State specific impacts associated with the incidence of empty trucks, noting that the positive parameter estimates for five States (New South Wales, Queensland, Western Australia, South Australia, Victoria), compared to Tasmania and the Northern Territory, have systematic differential impacts on the likelihood of empty truck volumes. We know that there are a lot of directional impacts associated with the lack of backhaul business, and this is greater in the larger populated States, with evidence that there is a higher tendency in the lesser populated States to ensure there is a backhaul load before agreeing to undertake a distribution activity which often is long distance, as in the Northern Territory. But also, there are far more trucks available in the network in the larger States which adds to the risk of empty truck movements. The results also indicate that OD pairs associated with greater populations are less likely to be associated with empty truck movements. This is plausible since the amount of demand ensures that vehicles are better utilised, even if there is a high level of competing business activity available to move commodities. The predicted probability of empty truck movements, rigid and articulated, at an OD level for each commodity, is introduced into the truck type choice model as discussed below. This should be thought of as similar to a kilo tonnes trip except that they are zero kilo tonnes, and in that sense it is like a ‘commodity class’, and introduced into the aggregate truck type share model to remove the bias attributed to otherwise failing to account for movements that do not carry any commodities. Another way of saying this is that it is a class of movement which, as we have shown in Table 5, is influenced by exogenous factors, and if not accounted for would result in all predicted truck movements being associated with non-zero commodity flows.

## Aggregate truck type share model

The next model in the system is the multinomial logit model describing aggregate truck type shares, with results summarised in Table 6. The dependent variable is aggregated truck movements per annum by type between each OD pair for each commodity class.^15^ Frequencies are a transformation of proportions or shares, and hence are equivalent. The log likelihood function associated with the model is given as Eq. (6).

6 $$\sum\limits_{i = 1}^{N} {} \sum\limits_{j = 1}^{J} {\mathop W\nolimits_{i} } \mathop F\nolimits_{ij} LogP(i,j)$$

**Table 6:** Model results for the aggregate truck movement shares per annum by type between each OD pair for each commodity class (including zero commodity on board) for each of rigid and articulated trucks

Variables	Acronyms	Units	Parameter Estimates	t-value
*Rigid Truck*				
Constant	RIGIDASC		− 0.7777	− 463.11
Total annual kilo-tonnes	BSUMKTR	Kilo-tonne	0.0004	10.73
Kilo-tonne residuals	POIRESR	Kilo-tonne^2	− 0.0000005	− 7.95
Probability of truck being empty (no commodity flows)	CEMPTY1	Probability	0.5399	75.91
Live Animals	BLIVEANI	Dummy (1/0)	2.1010	510.32
Beverages and tobacco	BBEVTOB	Dummy (1/0)	0.8680	199.68
Crude materials	BCRUDE	Dummy (1/0)	0.7405	371.71
Cork and wood	BCRKWOOD	Dummy (1/0)	0.6365	317.72
Petroleum and petroleum products	BPETROL	Dummy (1/0)	0.7271	299.29
Chemicals	BCHEM	Dummy (1/0)	0.8680	334.51
Other manufactured articles	BOTHMANU	Dummy (1/0)	0.9494	113.64
Machinery and transport equipment	BMACHTP	Dummy (1/0)	0.8831	471.98
Sydney is the origin	BOSYD	Dummy (1/0)	1.5112	1189.03
Travel time (hours) between origin–destination	BTIME	Hour	− 1.2341	− 818.91
*Articulated Truck*				
Total annual kilo-tonnes	BSUMKTA	Kilo-tonne	0.0011	28.89
Kilo-tonne residuals	POIRESA	Kilo-tonne^2	− 0.0000009	− 13.59
Probability of truck being empty (no commodity flows)	CEMPTY2	Probability	0.2345	28.67
Cereal grains	CCGRAIN	Dummy (1/0)	− 1.0267	− 314.32
Food (animal or human consumption)	CFOOD	Dummy (1/0)	− 0.5710	− 291.34
Metalliferous ores and metal scrap	CMETORE	Dummy (1/0)	− 1.3220	− 240.48
Sand, stone and gravel	CSAND	Dummy (1/0)	− 0.1570	− 67.62
Tools of trade	CTTRADE	Dummy (1/0)	− 0.5734	− 200.94
Cement and concrete	CCEMCON	Dummy (1/0)	0.1428	52.26
Iron and steel	CIRONSTL	Dummy (1/0)	− 2.5856	− 271.3
Miscellaneous manufactured articles	CMISC	Dummy (1/0)	0.1158	43.69
Other commodities	COTHER	Dummy (1/0)	1.7505	552.98
Western Australia is the destination	BDWA	Dummy (1/0)	− 8.8069	− 283.44
South Australia is the destination	BDSA	Dummy (1/0)	− 1.2992	− 97.41
Travel time (hours) between origin–destination	BTIME	Hour	− 1.2341	− 818.91
**Model fit**				
McFadden Pseudo-R^2^	0.173			
Sample size	17,419			
AIC/N	1,551.80			

W_i_ is a weight, and F_ij_ is a frequency. For frequency data W_i_ = 1 and F_ij_ = the frequencies (or W_i_ = F_i1_ + F_i2_) and F_ij_ = Freq(i,j)/W_i_ equal to a proportion. The model estimation process converts these frequencies to predicted choice probabilities or shares, which are calibrated by additional commodity-specific constants (see below) to reproduce the observed vehicle type shares at a commodity level.

The overall goodness-of-fit is in line with what most non-linear discrete choice models obtained (Hensher et al. 2015), which in our case is 0.173. The signs of the estimated coefficients match a priori expectations. The statistically significant variables, in addition to commodity class-specific dummy variables, include total annual kilo-tonnes carried by each truck type between each OD pair, the average travel time between the centroids of each origin and destination as a generic parameter,^16^ a Sydney-specific origin dummy variable in the utility expression for rigid trucks, which is positive; and two destination-specific dummy variables for Western Australia and South Australia included in the utility expression for articulated trucks, which are negative influences of the probability of truck movements to these two States compared to other States.

We initially included the trip operating cost, but it was found to be highly correlated with travel time (given little congestion in many jurisdictions) and hence time and cost are both correlated with distance. We then used a generalised cost (or generalised time) specification converting cost or time to common units using a value of travel time savings. This also did not provide a meaningful result, being statistically insignificant, and so we have selected to use travel time only, which is essential when the model system is built into Metroscan with travel time the equilibrium assignment criterion. For subsequent use of the model system in Metroscan, where a scenario is a cost change (e.g., a carbon tax, congestion pricing, fuel changes), we developed a conversion between operating cost and travel time for each class of vehicle using the standard relationship between operating cost (in cents/km) and speed (in kilometres per hour).^17^ See Appendix C.

The control function correction is used to test for and correct if appropriate, endogeneity associated with kilo-tonnes (Table 4) and empty truck movements (Table 5) in the aggregate truck type share model. It is simply an auxiliary variable to correct for the endogeneity (Guevara et. al., 2015), so it is important to also include the exogenous variables that will be considered in the aggregate truck type share model, otherwise the control function correction may confound the effect of the exogenous variable with that of the residual. For the Poisson regression the *generalized residual* for kilo tonnes *is r*_*qi*_ = *Y(i)—Exp(beta'x(i))* = *Observed(i)—Expected(i)*, and for the aggregate logit share model for empty vs non-empty truck movements, the residuals are Y (1,0) minus the predicted choice probability.

To test for endogeneity associated with kilo-tonnes, the residuals of both ZIP models for vehicle kilo-tonnes were also used as an explanatory variable for the aggregate truck type share model as a way of purging the model of endogeneity bias associated with the specific variables. The squared residuals were included in the aggregate truck type share model. The residual variables are statistically significant but with relatively small effect sizes; so while it is appropriate to include them to correct for endogeneity, the expected impact is likely to be minimal, suggesting that the exogeneity assumption may not be problematic.^18^ The variable representing the probability of a truck being empty, obtained from the model reported in Table 5, is an estimate. The sign is positive, suggesting that, all other influences held constant, when the probability that a truck movement is empty compared to not being so, the probability has a differential impact on the share of truck movements that are rigid and articulated. The residual associated with the empty truck movement model was not statistically significant. Together with the estimated kilo-tonnes moved, this is an estimate, and hence to correct the estimated asymptotic covariance matrix for the randomness of the estimators carried forward, the standard Murphy and Topel (1985) correction is implemented, so that the standard errors are asymptotically efficient.

The dummy variables for commodity classes represent all classes; however, some commodities from the classes in Table 3 had statistically insignificant parameter estimates, due mainly to the relatively small amount of commodity being transported. The commodity class dummy variables were included in the utility expressions for either the rigid or articulated truck alternatives as a way of allowing for the role, on average, that the underlying characteristics of commodities in a class have on the preference for rigid or articulated trucks. They are effectively a decomposition of the alternative-specific constant to account for unobserved heterogeneity differences amongst the commodity classes that influence the predicted share of truck movements by vehicle type.

Elasticity estimates are obtained for two of the key variables of particular interest, travel time and annual kilo-tonnes. The direct elasticity of the aggregate share of truck movement by rigid trucks with respect to travel time between each OD pair across all commodity classes is -1.30. The equivalent estimate for articulated trucks is -0.62. These estimates are quite plausible, and we would expect a greater sensitivity for rigid vehicles because of the typical value of commodities. For kilo-tonnes carried between each OD pair across all commodity classes, the direct elasticities are -2.44 for rigid trucks and -0.62 for articulated vehicles. We have relatively elastic responses for rigid trucks and relatively inelastic responses for articulated vehicles. There is a greater sensitivity for rigid vehicles to changes in kilo-tonnes carried than the actual travel times, which suggests that volume is especially important to the truck movement business.

The next task is to calibrate the aggregate truck type share model to reproduce, at an OD level, the share of truck movements associated with each commodity class. The calibration procedure is straightforward (see Train 2009, p. 37) and initially involves adjusting the overall constant for each truck type (initially set at zero for articulated trucks), after accounting for the influence of all the variables, by a new parameter which is the original estimated alternative-specific constant plus, for each commodity class, the natural logarithm of the ratio of the observed share of truck movements in the reference year to the predicted share. The predicted shares are compared to the actual shares,^19^ and the constants are adjusted up or down until there is a match of the actual and predicted shares. Once the aggregate shares are reproduced through calibration, and checked through the assignment mechanism, we then looked at each and every OD pair and added an additional calibration constant where the observed and predicted shares deviate by more than 5%. There were very few OD pairs that required additional calibration. The final calibrated constants at the aggregated level (i.e., for all OD pairs) are summarised in Table 7 with the OD specific additional constants available on request.

**Table 7:** Calibrated constants for commodity types for the aggregate truck type share model

Commodity types	Initial constant (rigid)	Initial constant (artic)	Calibrated constant (rigid)	Calibrated constant (artic)	Share (rigid)	Share (artic)
Cereal grains	0.0000	− 1.0267	0.3811	0.8450	0.39	0.61
Food (animal or human consumption)	0.0000	− 0.5710	0.3138	0.3375	0.49	0.51
Live animals	2.1010	0.0000	1.7543	1.2223	0.63	0.37
Beverages and tobacco	0.8680	0.0000	0.8588	0.0215	0.70	0.30
Crude materials	0.7405	0.0000	0.7210	0.0397	0.66	0.34
Metalliferous ores and metal scrap	0.0000	− 1.3220	0.7624	0.9584	0.45	0.55
Sand, stone and gravel	0.0000	− 0.1570	− 0.2085	0.3061	0.37	0.63
Cork and wood	0.6365	0.0000	0.4864	0.2338	0.56	0.44
Tools of trade	0.0000	− 0.5734	0.1432	0.4826	0.42	0.58
Petroleum and petroleum products	0.7271	0.0000	0.5644	0.2705	0.57	0.43
Chemicals	0.8680	0.0000	0.8973	− 0.0735	0.73	0.27
Cement and concrete	0.0000	0.1428	− 0.5574	0.2579	0.31	0.69
Iron and steel	0.0000	− 2.5856	1.3240	2.3526	0.26	0.74
Other manufactured articles	0.9494	0.0000	1.0729	− 0.4149	0.82	0.18
Machinery and transport equipment	0.8831	0.0000	0.9081	− 0.0633	0.73	0.27
Miscellaneous manufactured articles	0.0000	0.1158	− 0.0953	− 0.0186	0.48	0.52
Other commodity	0.0000	1.7505	− 2.2601	0.0670	0.09	0.91

We compared the model predicted shares over the actual shares of the two types of trucks before and after the calibration of the constants. The average OD based prediction accuracy has been greatly improved, with the average probability prediction error reduced from − 0.091 to − 0.038 for articulated trucks and the average probability prediction error reduced from 0.117 to 0.041 for rigid truck by each OD pair.

It should be noted that the accuracy of predicted aggregate shares of rigid and articulated trucks are related to the available sample. For those commodities with very large sample size (e.g., food with 4651 observations), the accuracy of the predicted aggregate share is very high (i.e., the difference is as small as 1% to the actual share in the data). Some of the commodity types with a small sample of around 1000 also performed well (i.e., the accuracy is as close as 0.4% in the difference for a sample of 1294). The less accurate commodity types are often related to the limited sample size especially those below 1000 records in the dataset, such as iron and steel. We combined some of these commodities with very small sample sizes for the truck frequency models.

## Aggregate truck frequency model

A Poisson model for aggregate truck movements was estimated for each commodity type (19 models) with a constant term, a parameter estimate for logsum and the sigma (σ) term, where the latter parameter is the standard deviation of heterogeneity. The Poisson model without zero-inflation for each commodity class produced both good model fits and a high level of prediction accuracy, as shown in Table 8 for each model, since the ZIP parameter was not statistically significant. All parameters are statistically significant at the 1 percent level. The predicted frequency and actual frequency for each commodity type shows a high level of prediction accuracy with a regression R^2^ ranging from 0.81 to 0.94 for all commodity types, including commodity types with a relatively small sample size (e.g., general freight, see Table 2). The predicted truck movement frequencies for each row can almost perfectly predict the original truck movement frequency (R^2^ = 0.93 for actual and predicted frequencies). Including other candidate variables such as population, origin or destination dummies and interaction terms of logsums by commodity types, did not perform well. The prediction of the frequencies became different from the actual counts or negative and accuracy significantly dropped. We suggest this is because the tested variables are already significant explanatory influences on aggregate truck type shares and kilo-tonnes and hence are accommodated elsewhere in the overall model system. We also are aware of the risk of over-fitting and also of ensuring identification.

**Table 8:** Predicting total frequency of freight movement by commodity

Frequency of truck trips for commodity class and for empty trucks	Constant	Parameters for poisson model					Model fit and accuracy
		Logsum	t-value	Sigma	t-value	Sample	Predicted vs actual (R^2^)
Empty	8.7242	0.3358	***	1.2073	***	1484	0.92
Cereal grains	10.4410	0.4781	70,029.58	0.6049	28,637.59	154	0.88
Food (animal or human consumption)	6.3222	0.0102	10,606.14	1.6481	***	4651	0.84
Live animals	7.3521	0.4261	16,081.80	0.9597	11,037.12	1133	0.89
Beverages and tobacco	5.7992	0.2296	10,792.63	1.3260	11,760.21	1182	0.84
Crude materials	6.6850	0.0731	18,703.76	1.3616	58,142.47	3088	0.85
Metalliferous ores & metal scrap	6.7721	0.0866	4260.25	1.8984	85,843.17	365	0.81
Sand, stone and gravel	7.4166	0.2003	19,537.52	0.9956	25,209.24	1294	0.85
Cork and wood	6.5362	0.0267	12,574.66	1.7703	***	3695	0.81
Tools of trade	6.2435	0.0057	632.79	1.4247	31,860.60	1649	0.85
Petroleum and petroleum products	6.9336	0.1629	10,770.45	1.1338	23,240.14	786	0.88
Chemicals	6.2600	0.0440	4343.09	1.4466	40,033.78	2374	0.85
Cement and concrete	6.6774	0.4936	12,456.70	1.0033	15,532.08	1769	0.84
Other manufactured articles	4.7008	0.4449	1106.46	0.9775	850.98	540	0.87
Machinery and transport equipment	6.8607	0.0639	16,721.86	1.0488	27,718.94	3907	0.94
Miscellaneous manufactured articles	6.6564	0.5375	11,246.48	1.0926	21,234.95	1045	0.85
General freight	8.4175	0.0087	3709.75	0.7316	16,028.10	180	0.83
Other commodity	7.4547	0.4522	36,864.14	1.1833	66,291.72	481	0.92

Each row is a separately estimated model

Very large t-value is shown as “***”

**Fig. 2:**
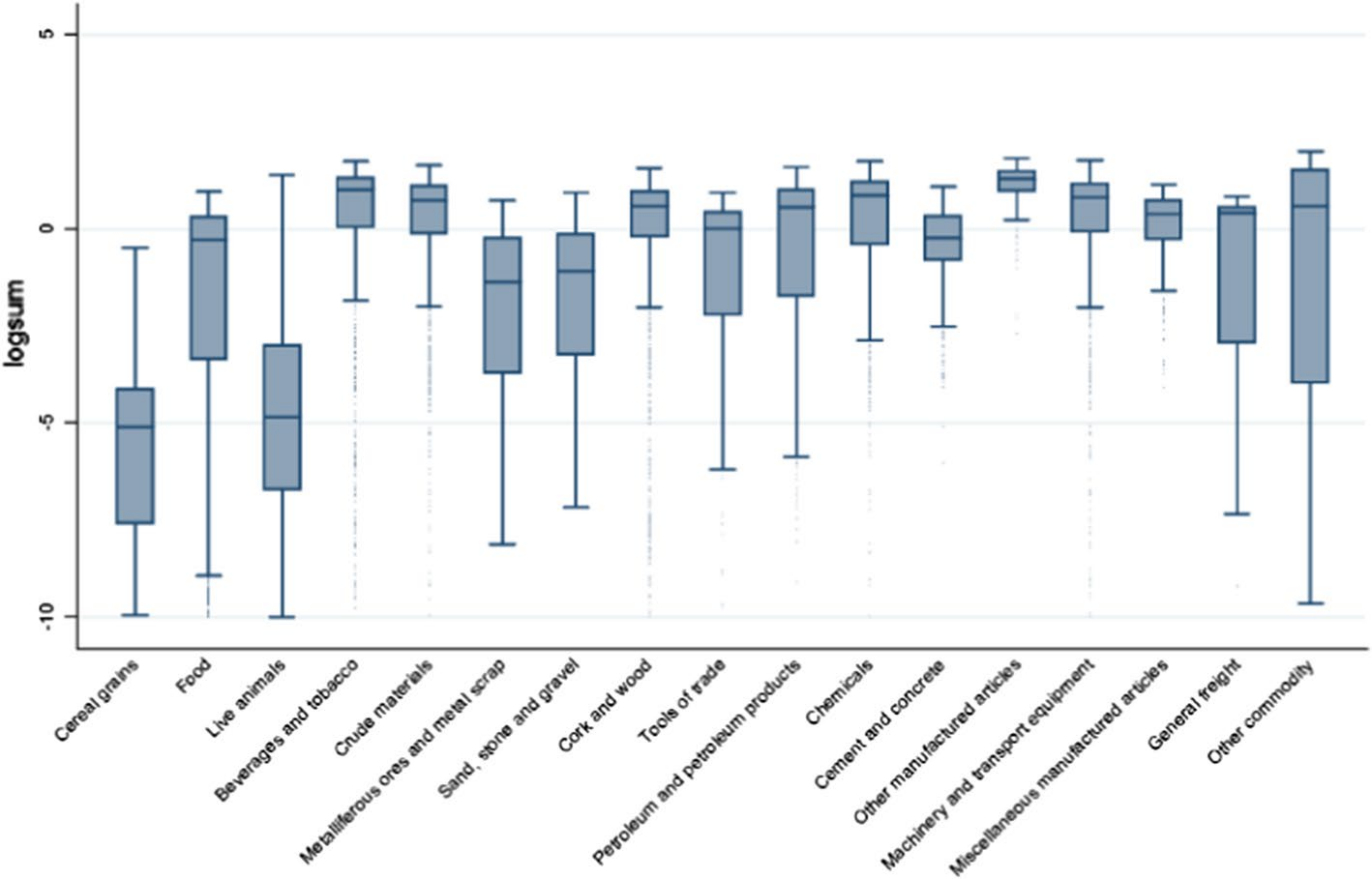
Logsums obtained by the aggregate truck type share model for each commodity class

The “other commodity” category combines the categories with very small sample sizes including coal, animal and vegetable oils, iron and steel and the original ‘other’ commodity categories. Figure 2 shows the distribution of predicted logsums across the commodity classes. The expected maximum utility (or logsum) varies a lot, as might be expected, between each commodity class, suggesting the influences on the choice between rigid and articulated trucks varies a great deal.^20^

## Application of the Freight models within MetroScan

In this section, we present and discuss simulated results and predictions made for three related transport scenarios, using the MetroScan system for the Greater Sydney Metropolitan Area (GSMA) in which the freight models are implemented^21^ (See Appendix A for the overall structure of MetroScan and Appendix B for the location of the GSMA). We illustrate the use of Metroscan with a distance based charge on trucks and cars, with a particular interest in identifying the importance of including freight activity with passenger activity in an integrated transport and land use model system. Appendix D provides a summary of other studies that have investigated distance based charges; although a word of caution is required in attempting the compare the evidence since contexts vary a great deal.

Scenario 1 assesses the impact in the transport sector and the wider economy of a distance based charge (DBC) introduced to passenger cars and trucks from the year 2021 onwards in the GSMA, at 5 cents per kilometre for cars and 20 cents per kilometre for trucks. Scenario 2 is related to Scenario 1, but only imposes a DBC on passenger cars at 5 cents per kilometre without a congestion charge for trucks. Scenario 3 is the opposite of Scenario 2, with a DBC only for trucks. These three scenarios enable us to identify the impact of a DBC when only passenger cars or trucks are assessed (i.e., we set the DBC to zero for the other mode), and when the DBC is imposed on both cars and trucks. We are unaware of this being considered in previous research. In the application through Metroscan, we obtain many outputs associated with efficiency, equity and environmental impact, including changes to passenger mode shares, truck movements, total kilometres and other measures.

## Background for the three scenarios

The idea of road pricing reform in transport is not new but has been a challenge politically for many years (Hensher and Puckett 2008; Hensher and Mulley 2014a, b; Hensher and Bliemer 2014, Bok et al. 2020a). While the debate has not gone away, there remains support from industry and academics for a revision to the way that cars and trucks are charged for the use of the road network, to reflect the true cost of usage, including the broader set of externalities such as emissions, safety and congestion. We propose a distance based charge for cars and trucks on all roads and at all times of the day in the GSMA. While variations could also be considered such as a peak period DBC and even a cordon-based charge in some locations, our interest herein is identifying the implications of a DBC on both freight and passenger vehicles in order to recognise that the costs and benefits of a DBC in one sector, passenger or freight, is impacted by policies implemented in the other sector; simply put because they share the same road network, and hence are affected by behavioural responses in both sectors (Moridpour et al. 2015).

Using Metroscan and introducing a DBC of 20 cents for trucks and 5 cents for passenger vehicles, we are able to predict the potential impact on traffic volumes through changes in freight and passenger trips, as well as changes in mode shares in both sectors. An important objective of road pricing reform is to improve the efficiency of the road network through reductions in traffic congestion as well as to achieve broader objectives linked to user benefits (firms and individuals) and emissions. An extensive number of outputs are obtained and reported as a way to identify the many ways in which a DBC impacts on users and the broader economy. Appendix C discusses how a DBC is introduced into the freight model system where there is no travel cost attribute.

The introduction of a distance based charge of road freight and passenger vehicles has varying impacts throughout the model system as is shown in the various output results in Table 9 (Scenarios 1–3). These changes occur throughout the interrelated model system in MetroScan; however, for example, when we introduce the DBC on trucks only, the passenger models greatest impacted are the vehicle kilometres model, linked to changes in travel times that are fed up from the mobility investment module (essentially mode and time of day for work and non-work trips). There are noticeable switches between car and public transport (see Scenario 3). The change in the freight model system is linked back to the change in travel time (and the conversion formula for generalised cost) which impacts on the aggregate truck type shares and the linked (via a logsum) aggregate truck movements. In addition, we have the traffic assignment that adjusts travel times etc., until we obtain an equilibrium outcome in respect of levels of freight movement for each commodity type and passenger vehicle movements for each OD pair.

**Table 9:** MetroScan outputs for the three scenarios

Scenario 1: Distance based charges from 2021 onwards at 5c/km for passenger cars and 20c/km for trucks: 2030 results					
Acronyms	Output Items	Units	Base	Project	Difference (%)
TCO2 (kg)	Total annual carbon dioxide all modes	Kilograms (kg)	9,802,478,595	8,695,693,958	− 11.29
TAIRPOL ($)	Total annual local air pollution costs	Dollars ($)	5,536,737,112	5,128,613,118	− 7.37
*Passenger car outputs*					
TCO2C (kg)	Total annual carbon dioxide for passenger cars	Kilograms (kg)	8,810,641,666	7,738,250,429	− 12.17
TAIRPOLC ($)	Total annual local air pollution costs for passenger cars	Dollars ($)	2,627,598,444	2,307,779,110	− 12.17
TEUCPVMC ($2019)	Total annual end-use money cost in present value terms (all trip purposes and all modes)	Dollars ($)	50,321,890,901	49,330,223,486	− 1.97
TEUCPVTTC ($2019)	Total annual end-use travel time cost in present value terms (all trip purposes and all modes)	Dollars ($)	70,733,133,316	67,032,701,369	− 5.23
TVKM (km)	Total annual passenger vehicle kilometres (all trip purposes for car)	Kms	77,970,280,229	68,480,092,289	− 12.17
Tenergy (litres)	Total annual energy consumed by cars	Litres	32,939,626	29,106,206	− 11.64
TGovtExcise ($)	Total annual government revenue fuel excise for cars	Dollars ($)	3,430,692,330	3,013,124,061	− 12.17
TRCongC ($)	Total annual revenue from congestion pricing	Dollars ($)	0	3,424,004,614	–
TPT ($)	Total annual revenue from public transport use	Dollars ($)	2,705,775,075	3,238,035,092	19.67
TDA (proportion)	Modal share for car drive alone all trip purposes	Proportion	0.7547	0.7526	− 0.28
TRS (proportion)	Modal share for rideshare all trip purposes	Proportion	0.1699	0.1565	− 7.87
Ttrain (proportion)	Modal share for train travel all trip purposes	Proportion	0.0430	0.0530	23.18
Tbus (proportion)	Modal share for bus travel all trip purposes	Proportion	0.0324	0.0379	17.02
TDARS + TRSPA	Total annual car trips (as driver and passenger)	Number	3,914,948,373	3,839,548,988	− 1.93
C02PVKM	CO_2_ per car kilometre	CO2/vkm	0.24	0.24	1.41
GCPersT ($)	Generalised cost per person trip for car	$/car person trip	23.93	22.94	− 4.13
GCPubT ($)	Generalised cost per person trip for public transport	$/PT person trip	20.19	20.42	1.17
TEUGCPersT ($)	Total end use generalised cost	$/person trip	23.59	22.67	− 3.90
EMUDTMCPersT ($)	Departure Time and Mode Choice Consumer surplus per person trip	$/person trip	1.562	− 0.249	− 115.94
EMURLCPersT ($)	Residential Location (total) Consumer surplus per person trip	$/person trip	− 0.023	− 0.039	− 69.57
*Freight outputs*					
TCO2T (kg)	Total annual carbon dioxide for trucks	Kilograms (kg)	991,836,929	957,443,529	− 3.47
TAIRPOLT ($)	Total annual local air pollution costs for trucks	Dollars ($)	2,909,138,669	2,820,834,008	− 3.04
movefreqR	Total annual movement frequency for rigid trucks	Number	17,205,451	16,424,082	− 4.54
movefreqA	Total annual movement frequency for articulated trucks	Number	18,933,805	18,403,459	− 2.80
TVKMR	Total annual distance travelled by rigid trucks	Kms	2,374,352,183	2,266,523,338	− 4.54
TVKMA	Total annual distance travelled by articulated trucks	Kms	3,824,628,621	3,717,498,720	− 2.80
TRCongT ($)	Total annual revenue from congestion pricing	Dollars ($)	0	1,196,804,412	–
FRTEUCPVMC ($2019)	Total annual end-use money cost in present value terms	Dollars ($)	3,746,198,494	3,618,158,872	− 3.42
FRTEUCPVTTC ($2019)	Annual total end-use travel time cost in present value terms	Dollars ($)	4,179,515,080	4,036,665,326	− 3.42
FRTEMUDTMC ($)	Total annual expected maximum utility for aggregate truck type share	Dollars ($)	5,588,111,261	4,073,364,426	− 27.11
FRTGovtExcise($)	Total annual government revenue from fuel excise	Dollars ($)	1,202,602,276	1,160,900,280	− 3.47

Scenario 2: Distance based charges from 2021 onwards at 5c/km for passenger cars: 2030 results					
Acronyms	Output Items	Units	Base	Project	Difference (%)
TCO2 (kg)	Total annual carbon dioxide all modes	Kilograms (kg)	9,802,478,595	8,605,960,204	− 12.21
TAIRPOL ($)	Total annual local air pollution costs	Dollars ($)	5,536,737,112	5,215,300,398	− 5.81
*Passenger car outputs*					
TCO2C (kg)	Total annual carbon dioxide for passenger cars	Kilograms (kg)	8,810,641,666	7,599,954,323	− 13.74
TAIRPOLC ($)	Total annual local air pollution costs for passenger cars	Dollars ($)	2,627,598,444	2,266,535,050	− 13.74
TEUCPVMC ($2019)	Total annual end-use money cost in present value terms (all trip purposes and all modes)	Dollars ($)	50,321,890,901	50,261,135,105	− 0.12
TEUCPVTTC (42,019)	Total annual end-use travel time cost in present value terms (all trip purposes and all modes)	Dollars ($)	70,733,133,316	67,635,309,569	− 4.38
TVKM (km)	Total annual passenger vehicle kilometres (all trip purposes for car)	Kms	77,970,280,229	67,256,232,949	− 13.74
Tenergy (litres)	Total annual energy consumed by cars	Litres	32,939,626	28,660,254	− 12.99
TGovtExcise ($)	Total annual government revenue fuel excise for cars	Dollars ($)	3,430,692,330	2,959,274,250	− 13.74
TRCongC ($)	Total annual revenue from congestion pricing	Dollars ($)	0	3,362,811,647	–
TPT ($)	Total annual revenue from public transport use	Dollars ($)	2,705,775,075	3,362,572,216	24.27
TDA (proportion)	Modal share for car drive alone all trip purposes	Proportion	0.7547	0.7500	− 0.63
TRS (proportion)	Modal share for rideshare all trip purposes	Proportion	0.1699	0.1556	− 8.41
Ttrain (proportion)	Modal share for train travel all trip purposes	Proportion	0.0430	0.0552	28.21
Tbus (proportion)	Modal share for bus travel all trip purposes	Proportion	0.0324	0.0393	21.33
TDARS + TRSPA	Total annual car trips (as driver and passenger)	Number	3,914,948,373	3,823,300,980	− 2.34
C02PVKM	CO_2_ per car kilometre	CO2/vkm	0.24	0.24	2.00
GCPersT ($)	Generalised cost per person trip for car	$/car person trip	23.93	23.21	− 2.99
GCPubT ($)	Generalised cost per person trip for public transport	$/PT person trip	20.19	20.58	1.96
TEUGCPersT ($)	Total end use generalised cost	$/person trip	23.59	22.92	− 2.84
EMUDTMCPersT ($)	Departure Time and Mode Choice Consumer surplus per person trip	$/person trip	1.562	− 0.527	− 133.74
EMURLCPersT ($)	Residential Location (total) Consumer surplus per person trip	$/person trip	− 0.023	− 0.042	− 82.61
*Freight outputs*					
TCO2T (kg)	Total annual carbon dioxide for trucks	Kilograms (kg)	991,836,929	1,006,005,880	1.43
TAIRPOLT ($)	Total annual local air pollution costs for trucks	Dollars ($)	2,909,138,669	2,948,765,348	1.36
movefreqR	Total annual movement frequency for rigid trucks	Number	17,205,451	17,479,626	1.59
movefreqA	Total annual movement frequency for articulated trucks	Number	18,933,805	19,184,893	1.33
TVKMR	Total annual distance travelled by rigid trucks	Kms	2,374,352,183	2,412,188,448	1.59
TVKMA	Total annual distance travelled by articulated trucks	Kms	3,824,628,621	3,875,348,304	1.33
TRCongT ($)	Total annual revenue from congestion pricing	Dollars ($)	0	1,257,507,350	–
FRTEUCPVMC ($2019)	Total annual end-use money cost in present value terms	Dollars ($)	3,746,198,494	3,799,428,453	1.42
FRTEUCPVTTC ($2019)	Annual total end-use travel time cost in present value terms	Dollars ($)	4,179,515,080	4,238,902,060	1.42
FRTEMUDTMC ($)	Total annual expected maximum utility for aggregate truck type share	Dollars ($)	5,588,111,261	6,117,533,743	9.47
FRTGovtExcise($)	Total annual government revenue from fuel excise	Dollars ($)	1,202,602,276	1,183,188,666	1.43

Scenario 3: Distance based charges from 2021 onwards at 20c/km for trucks: 2030 results					
Acronyms	Output Items	Units	Base	Project	Difference (%)
TCO_2_ (kg)	Total annual carbon dioxide all modes	kilograms (kg)	9,802,478,595	9,961,765,508	1.66
TAIRPOL ($)	Total annual local air pollution costs	Dollars ($)	5,536,737,112	5,494,768,925	− 0.76
*Passenger car outputs*					
TCO2C (kg)	Total annual carbon dioxide for passenger cars	kilograms (kg)	8,810,641,666	9,012,401,723	2.29
TAIRPOLC ($)	Total annual local air pollution costs for passenger cars	Dollars ($)	2,627,598,444	2,687,769,363	2.29
TEUCPVMC ($2019)	Total annual end-use money cost in present value terms (all trip purposes and all modes)	Dollars ($)	50,321,890,901	49,537,479,488	− 1.56
TEUCPVTTC ($2019)	Total annual end-use travel time cost in present value terms (all trip purposes and all modes)	Dollars ($)	70,733,133,316	70,458,568,861	− 0.39
TVKM (km)	Total annual passenger vehicle kilometres (all trip purposes for car)	kms	77,970,280,229	79,755,767,462	2.29
Tenergy (litres)	Total annual energy consumed by cars	Litres	32,939,626	33,611,630	2.04
TGovtExcise ($)	Total annual government revenue fuel excise for cars	Dollars ($)	3,430,692,330	3,509,253,768	2.29
TRCongC ($)	Total annual revenue from congestion pricing	Dollars ($)	0	0	–
TPT ($)	Total annual revenue from public transport use	Dollars ($)	2,705,775,075	2,601,666,764	− 3.85
TDA (proportion)	Modal share for car drive alone all trip purposes	Proportion	0.7547	0.7556	0.11
TRS (proportion)	Modal share for rideshare all trip purposes	Proportion	0.1699	0.1720	1.24
Ttrain (proportion)	Modal share for train travel all trip purposes	Proportion	0.0430	0.0413	− 4.12
Tbus (proportion)	Modal share for bus travel all trip purposes	Proportion	0.0324	0.0312	− 3.66
TDARS + TRSPA	Total annual car trips (as driver and passenger)	Number	3,914,948,373	3,928,943,555	0.36
C02PVKM	CO_2_ per car kilometre	CO_2_/vkm	0.24	0.23	− 0.55
GCPersT ($)	Generalised cost per person trip for car	$/car person trip	23.93	23.78	− 0.64
GCPubT ($)	Generalised cost per person trip for public transport	$/PT person trip	20.19	20.07	− 0.60
TEUGCPersT ($)	Total end use generalised cost	$/person trip	23.59	23.45	− 0.58
EMUDTMCPersT ($)	Departure Time and Mode Choice Consumer surplus per person trip	$/person trip	1.562	1.894	21.25
EMURLCPersT ($)	Residential Location (total) Consumer surplus per person trip	$/person trip	− 0.023	− 0.019	17.39
*Freight outputs*					
TCO2T (kg)	Total annual carbon dioxide for trucks	kilograms (kg)	991,836,929	949,363,785	− 4.28
TAIRPOLT ($)	Total annual local air pollution costs for trucks	Dollars ($)	2,909,138,669	2,806,999,561	− 3.51
movefreqR	Total annual movement frequency for rigid trucks	Number	17,205,451	16,138,993	− 6.20
movefreqA	Total annual movement frequency for articulated trucks	Number	18,933,805	18,348,231	− 3.09
TVKMR	Total annual distance travelled by rigid trucks	kms	2,374,352,183	2,227,181,078	− 6.20
TVKMA	Total annual distance travelled by articulated trucks	kms	3,824,628,621	3,706,342,576	− 3.09
TRCongT ($)	Total annual revenue from congestion pricing	Dollars ($)	0	1,186,704,731	–
FRTEUCPVMC ($2019)	Total annual end-use money cost in present value terms	Dollars ($)	3,746,198,494	3,589,104,730	− 4.19
FRTEUCPVTTC ($2019)	Annual total end-use travel time cost in present value terms	Dollars ($)	4,179,515,080	4,004,250,540	− 4.19
FRTEMUDTMC ($)	Total annual expected maximum utility for aggregate truck type share	Dollars ($)	5,588,111,261	3,508,952,052	− 37.21
FRTGovtExcise($)	Total annual government revenue from fuel excise	Dollars ($)	1,202,602,276	1,151,103,589	− 4.28

The generalised cost per person trip per for car (GCpersT) and generalised cost per person trip for public transport (GCPubT) are given as follows: GCpersT = VTTS*in-vehicle time + VoR*buffer time + operating cost ($/trip) + tollcost ($/trip) for all purpose of trips (peak/offpeak); and GCPubT = invt VTTS *invehicle travel time + out-of-vehicle VTTS *out of vehicle travel time + PT fare ($/trip) for all purpose of trips(peak/offpeak)

All trip purposes = commuting, non-commuting and business Base year $2019

## Impact on the transport system

Table 9 summarises, for the three scenarios, a number of informative output results for 2030, noting that Metroscan generates forecasts of all such outputs for each year from 2021 onwards up to 2056. The DBC is set to start from 2021 for the three scenarios, with 2030 being an appealing year in which to show ongoing and consistent impact of the DBC policy.

The total distance travelled of rigid trucks (TVKMR), and articulated trucks (TVKMA) under a DBC has the largest percentage reduction at -6.20% and -3.09% respectively for Scenario 3 when the DBC is applied for trucks only. This results in a 2.29% increase in car kilometres (TVKM), clearly due to improved travel conditions where there are less truck kilometres in the network. In contrast, for a DBC associated with cars only (Scenario 2), we see a 13.74% reduction in total annual car kilometres but a 1.59% increase in rigid truck kilometres and a 1.33% increase for articulated truck kilometres. When we assess the impact of a DBC applied to all road modes (Scenario 1), we see a 12.17% decrease in car kilometres, a 4.54% reduction in rigid truck kilometres, and a 2.8% decrease for articulated trucks. All of these findings reinforce the importance of not focussing on one modal segment since the flow-on effects across all road activity are significant and will have implications for the economic appraisal and impact of the road pricing reform agenda. Even if we only focussed on a DBC for cars, the impact on truck movements needs to be recognised (at 1.59% under Scenario 2).

A related impact is on travel time, defined as the total end-use travel time in present value terms (TEUCPV_TTC). For a DBC on only cars (Scenario 2), there is a 4.38% improvement in travel time, which when combined with a DBC for trucks (Scenario 1), improves further to 5.23%, given a gain of 3.42% for trucks. This is an expected result given the dominance of cars in the road network. If only trucks were subject to a DBC (Scenario 3), the travel time benefit for cars improves by only 0.39%; however, trucks obtain a 4.19% increase in benefit.

While the impact of a DBC delivers improved travel times and contributes to reducing traffic congestion, it also has desirable environmental benefits. We see an 11.29% reduction in CO_2_ emissions when the DBC is imposed on both cars and trucks, with 12.17% reduction associated with cars and 3.47% reduction for trucks. These are sizeable reductions in enhanced greenhouse gas emissions. With a single sector DBC, we would see CO_2_ increasing in the sector not subject to a DBC, with car increasing by 2.29% when a DBC is introduced for trucks only and 1.43% for trucks when the DBC is applied to cars only. The overall impact on total energy consumed (in litres of fuel) is also significant and aligned with CO_2_ changes given their common link to fuel efficiency, with a 12.99% reduction for cars under a DBC applied only to cars, which reduces to 11.64% when a DBC is also imposed on trucks.

The implications on truck movements are of special interest given the model framework presented above. When a DBC is applied to cars and trucks, we see a 4.54% and a 2.8% reduction respectively in rigid (movefreqR) and articulated (movefreqA) truck movements, which is adjusted up to 6.2% and 3.09% respectively when the DBC is only imposed on trucks. This again shows the interdependencies between cars and trucks regardless of whether one or both are subject to the DBC policy instrument.

The implications of this evidence flow through to a number of other output measures. For example, when the DBC is imposed on both sectors, we see a decrease in government fuel excise of 12.17% from cars and 3.47% from trucks; but this is compensated by a significant increase in revenue from the DBC, being $11.972 m per day (or approximately $3.424 billion per annum) for cars and 1.197 billion annual revenue from trucks. This a sizeable revenue stream, close to $4.6 billion per annum. Another informative output indicator is the consumer surplus change consequent on DBC. Holding residential and firm location fixed, and allowing for modal and time of day switching responses for all passengers, we see an average loss of consumer surplus benefit of 15.92% per person trip for passenger trips by all modes under a DBC in both sectors, but an increase on average of 21.25% when the DBC is applied only to trucks, and a 33.74% reduction when only applied to cars. It should be noted that these percentage changes are associated with numerically small absolute levels of consumer surplus before and after the introduction of a DBC.

In the passenger sector, when we have a DBC only on car kilometres, the mode shares for drive alone (TDA) and cars with passengers (TRS) reduce by 0.63% and 8.41% respectively, while the shares for train and bus increase by 28.21 and 21.33 respectively for train and bus, noting that public transport has a relatively small share (7.74%) of the overall travel movements. When we impose a DBC on cars and trucks, the reduction in percentage changes is 0.28, 7.87 respectively for car drive alone and car with passengers, which is lower than for a car only DBC, which is expected given the improvement associated with fewer truck kilometres. This also results in a drop in the percentage share for train and bus, now 23.18 and 17.02 percent respectively. This translates into a healthy increase in public transport revenue of 19.67% under a DBC for both sectors and 24.27% when the DBC is applied to cars only (where there is a greater switch into public transport). This is another important finding indicating that the switch into public transport is tempered when we account for pricing reforms in both the passenger and freight sectors.

There are significantly different contributions to local air pollution for passenger cars and trucks, especially heavy articulated trucks. While the suggested cost per kilometre recommended by TfNSW (2020) for passenger cars is only 3.37 cents/km, the suggested air pollution costs are as high as 16.5 cents/km for rigid trucks and 65.82 cents/km for articulated trucks. The impact of changes in total kilometres for cars and trucks can result in different patterns for CO_2_ emissions and local air pollution. The combined change in CO_2_ from cars and trucks under the various DBC scenarios is closer to the level of change in cars attributed to the smaller difference in CO_2_ emissions from cars and trucks and much larger total kilometres by cars (i.e., 113 g/km for cars and 160 g/km for trucks on average for CO_2_ emission). In contrast, for air pollution, the air pollution from trucks accounts for more than 50% of air pollution in the land transport sector, and hence the relatively small change in truck kilometres can dominate a much more sizeable change in car kilometres. For example, when DBC is imposed on trucks only (Scenario 3), the reduction in air pollution for trucks is disproportionately greater than the increase in air pollution for cars, which is not the case for CO_2_.

## Conclusions

This paper has set out to develop a very practical and easy way to apply a model system for freight demand truck movements and to place the model system within a setting in which freight movement activity competes side by side with passenger transportation in the road network. The appeal is that it is simple to use, and captures the key linkages between the demand for commodities, the role of truck types in moving this volume, including zero commodity volume, and what this means for the overall amount of truck movement on the road network. Commodity demand and land use effects that change over time (given exogenous forecasts of influences such as population and employment) can be used to inform the freight movement task, and how it impacts on the overall performance of the transport system, beyond only freight activity.

The final set of calibrated freight demand models are inter-dependent, which is necessary for an integration into a unified, integrated transport and land use model system such as MetroScan that also recognises the interdependencies, hence endogeneity and feedback, between passenger and freight movement activity. Specifically, the kilo-tonnes model is a structural representation of the generation of volumes of commodities, including zero volume that have to be distributed and is influenced by population and employment in particular, which are proxies for economic activity. For a given quantum of kilo-tonnes which enters the aggregate truck type share model, we can predict the share of truck movements between each OD pair within a commodity class and for circumstances when truck movements do not carry any freight. The predicted aggregate truck type shares have an associated logsum variable that represents the expected maximum utility associated with the underlying preferences of the truck share model. The logsum is the key influence on the aggregate truck movements between each OD pair within each commodity class.

This aggregate truck movement in the base and application scenario feeds into the road traffic assignment module of MetroScan, and together with other road traffic, mainly passenger cars and light commercial vehicles, is used in establishing the equilibrium performance level in the road network.

Through iterations and feedback into the freight model system, where adjustments in travel times influence the changing truck shares and frequencies in the network, we arrive at a solution given the convergence criteria in the traffic assignment algorithm. The solution represents the travel times and the volume of truck movements between each OD pair within commodity class on the roads linking these geographical jurisdictions. We have demonstrated the rich policy capability of the Metroscan framework that embeds the freight model system with passenger and locational modules in order to study the implications of introducing a distance-based charging regime for trucks and cars. The key policy take away is that a pricing strategy be it on cars alone, trucks alone or both impacts of the performance and cost of each mode in the road network, and that both should be allowed for in an studying the impact of road priding reform.

In ongoing research we are enhancing the model system in two ways. Firstly, we will develop appropriate ways to recognise the changes in freight and passenger movements associated with the growth in e- (or online) shopping. At the time of model development and application, e-shopping in Australia was very small (less than 3%—see https://www.finder.com.au/online-shopping-statistics). These purchases will still have to be transported to warehouses or integrated logistics hubs or other locations as part of the supply chain and this will retain a large amount of freight distribution by trucks; however some freight will then be distributed by light commercial vehicles (LCVs) as part of the last mile. We need also to recognise and account for the growing collection of online orders from shops as part of click and collect, and hence the change in passenger trips will not be fully associated with online shopping. Currently, the growth of online shopping can be accommodated with the current version of MetroScan through scenario analysis by adjusting the amount of freight being delivered by light commercial vehicles (LCVs) as well as reductions in passenger trips. Secondly, we will investigate whether an additional model that considers the role of rail over road distribution for non-bulk commodities is warranted, given the dominance of non-bulk movements being by truck; however the initial focus on road freight distribution is important not only as the main way in which freight movement interacts with passenger movements in the land transport context, but trucks provide an important role in distributing the goods moved by coastal shipping, rail and air to and from the initial origins and final destinations, commonly referred to as the first and last mile.

## Appendix A: The metroscan structure


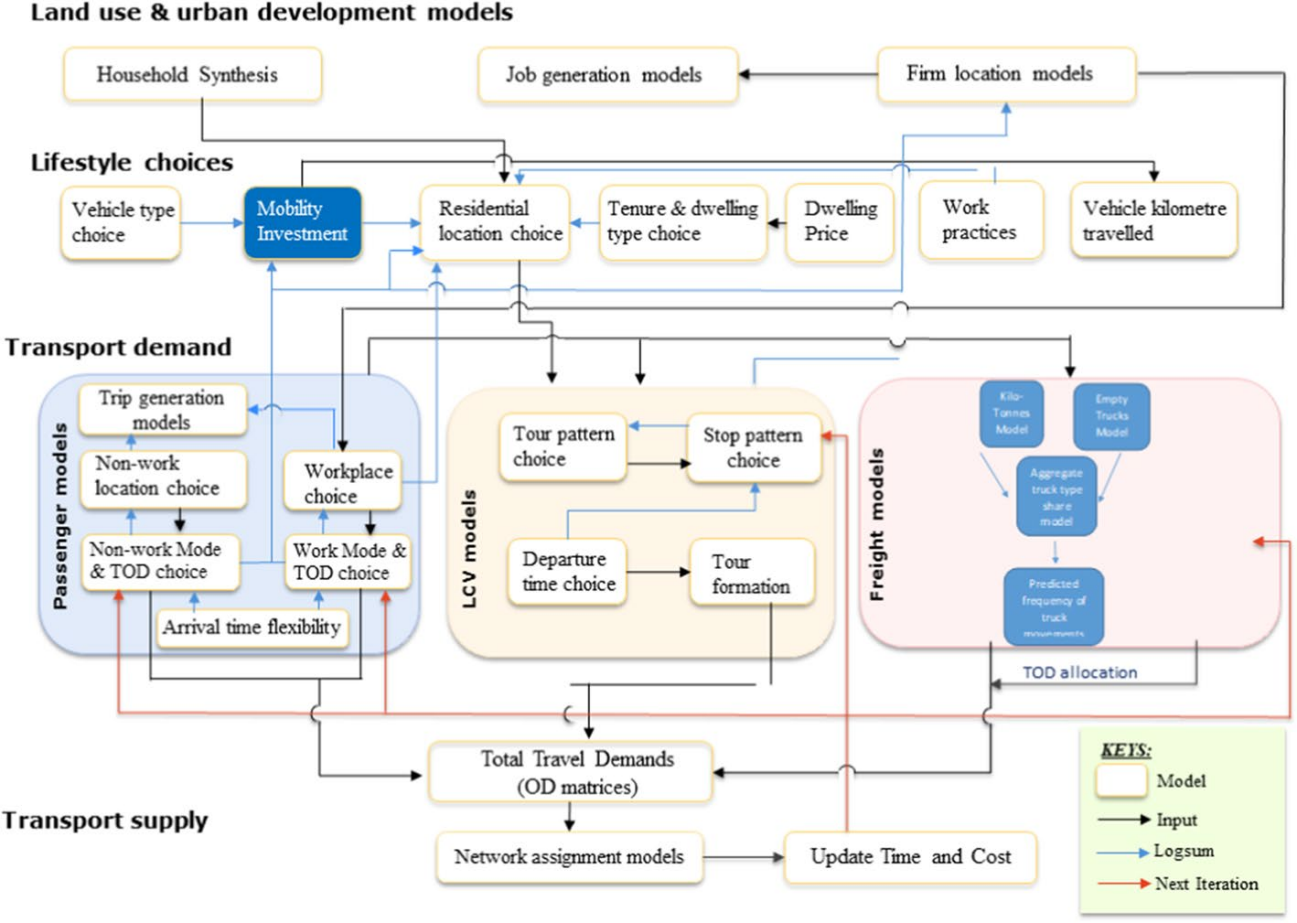
Source: Hensher et al. (2020a)

## Appendix B: Geographical locations for the greater Sydney Metropolitan Area (Newcastle, Sydney and Wollongong), and SA3 zones for both Sydney and Australia


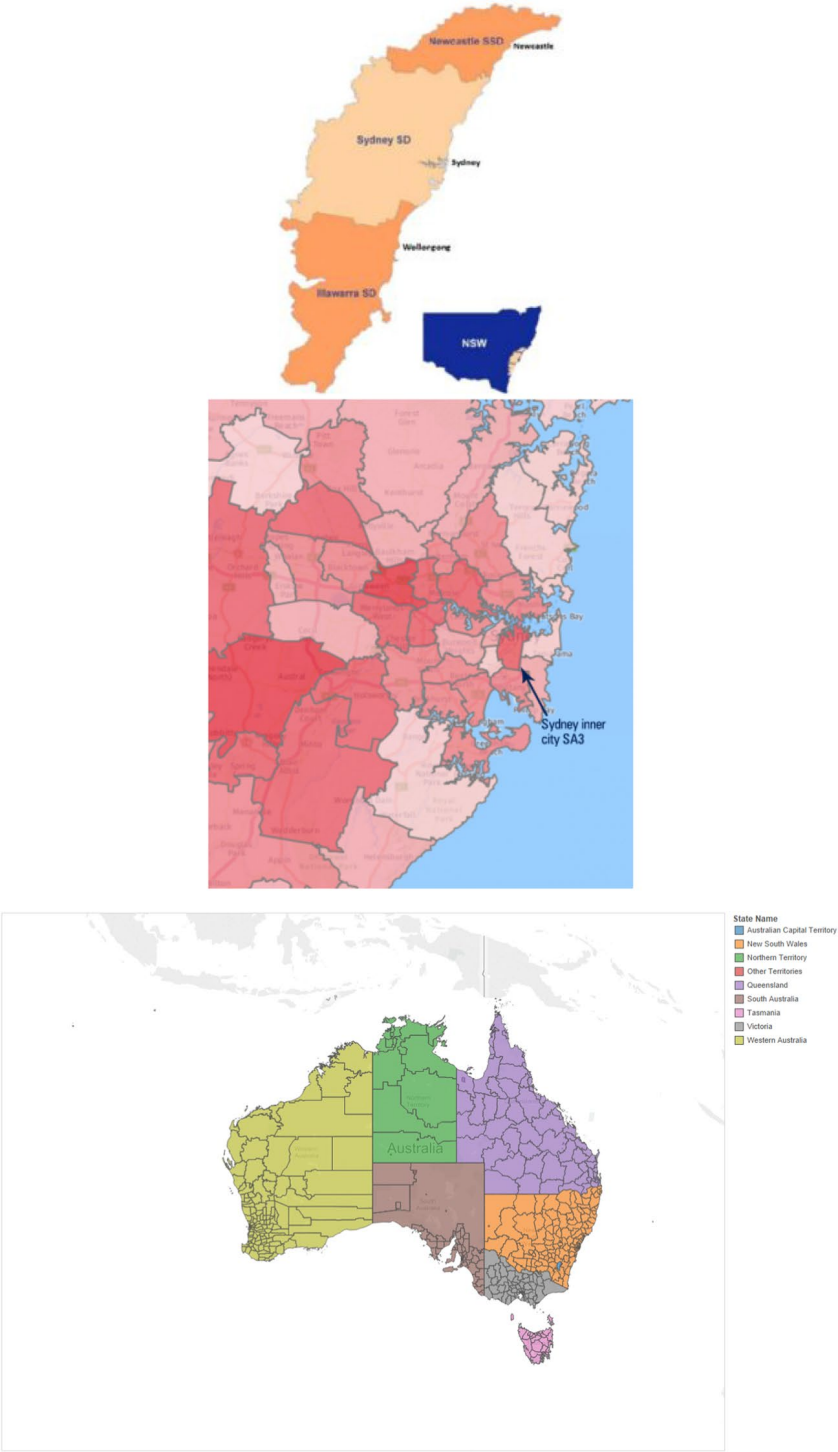


## Appendix C: Travel time and costs for rigid and articulated trucks

In the aggregate truck type share model, we have included the trip time as one of the variables to predict the aggregate truck type shares (“BTIME” in Table 6). In practice, we often need to examine the impact of transport cost instead of using trip time on truck movement. These changes may be related to changes in fuel, toll, excise rate or any other types of operation and maintenance costs. The impact for potential new policies such as extra charges due to emission control or congestion control are also highly likely for the future. Travel time and travel cost are closely related in many measures such as the Value of Time (VOT). Using widely accepted values for VOT such as the ones used in Benefit Cost Analysis (BCA) recommended by government departments, we have derived the coefficient for cost per hour for both truck types, using Eq. 7.

7 $$VOT = \beta_{time} /\beta_{cost}$$

We have used the commonly accepted VOT in Australia of $42.63/hour for rigid trucks and $64.72/hour for articulated trucks (TfNSW 2020) after also checking sources such as the Australian Transport Assessment and Planning (ATAP) and our past research. The parameters for the rigid and articulated trucks are -0.0296 and -0.0195 relatively, for transport costs in the dollar per hour unit.

To test a scenario with cost change, we can alter the part of a utility expression contributed by travel time for aggregate truck type share from *β*_time_*Time to *β*_cost_*VOT*(New/Current cost)*Time without changing the utility. To be specific, for rigid and articulated trucks, the related utility component related to time and cost can be given in two forms as below.

*Using time only:*

$${\text{Utility}}_{{{\text{rigid}}}} \; = \; \ldots \; - \;1.2618*{\text{time}}$$

$${\text{Utility}}_{{{\text{articulated}}}} \; = \; \ldots \; - \;1.2618*{\text{time}}$$

*Using cost and time:*

$${\text{Utility}}_{{{\text{rigid}}}} \; = \; \ldots \; - 0.0296*42.63*\left( {{\text{new}}/{\text{current}}\;{\text{costs}}} \right)*{\text{time}}$$

$${\text{Utility}}_{{{\text{articulated}}}} \; = \; \ldots \; - \;0.0195*64.72*\left( {{\text{new}}/{\text{current}}\;{\text{costs}}} \right)*{\text{time}}$$

When we implement the freight model in the MetroScan system, the car and truck interactions, such as travel time, were also linked, based on past research. For example, the travel time associated with trucks was linked with the travel time for cars using the following equation in Kim and Mahmassani (1978, p 37):

8 $${\text{Time}}_{{{\text{truck}}}} \; = \;\beta 0 \, \; + \;\beta 1*{\text{ Time}}_{{{\text{car}}}} \; + \;\varepsilon$$

In their model, β_0_ was -4.78 and β_1_ was 1.075, respectively. With these links built in, freight models could work cohesively with other models such as a passenger mode choice model within an integrated system to reflect changes in both passenger car and freight vehicle movements.

## Appendix D: Summary of a number of studies that have investigated distance-based charging

The research on either DBC or time and distance combined charges have been discussed by researchers from different countries such as Europe, US, Singapore and Australia. DBC policies have been implemented in some EU countries such as the Netherlands, with a main focus of research on optimising the DBC design or quantum (e.g., Bok et. al. 2021). In countries like Singapore where the transport authority has implemented cordon-based charges for the Central area, research has investigated combining distance-based and time-based charges into a simple cordon-based charge (e.g., Gu et. al., 2018; Liu et. al., 2014 and Meng et. al., 2012). In the USA and Australia where governments are facing inadequate funds for maintaining existing transport and road systems, studies have looked into ways of establishing an equitable and progressive DBC schemes to achieve multiple goals including raising adequate funds for road investment and maintenance while improving equity in these charges for both passengers and businesses (e.g., Hensher et. al. 2014; Yang et. al., 2014).

A summary of a number of studies that have investigated distance-based charging.

**Table:** 

Paper reference	Freight (F), Passenger (P)	DBC rate ($…)	Output measure	Impact
Bok et al. (2021)	F	5, 15 and 29 cent/km	Tonne kilometres	0.4–4.8% reduction
Hensher et al. (2014)	P	5c/km peak only, 50% registration fee	Car kilometres and financial gain	4.7% reduction in total annual peak period kilometres, and a 2.96% reduction in all kilometres The average annual financial gains and losses per driver at 5 cents/km are relatively small, ranging from $91 to -$40
Cavallaro et al. (2018)	P	Table 3 Euro 0.6/km (~ $1 AUD)	CO_2_ emissions	8% reduction
Gu et al. (2018)	Any	Ideal DBC at $1.05/km within the cordon area	Congestion	Congestion controlled
Lentzakis et al. (2020)	Any	Adaptive DBC and adaptive cordon-based charge S$0.13/km to S$0.96/km	Social welfare Consumer surplus Travel time	DBC with tolling zone definition performs better than cordon-based charge
Liu et al. (2014)	Any	Time and distance combined cordon-based toll in Singapore (e.g., $0.3/min for time plus $1, $2, and $3.5 for distance from 9–15 kms	Congestion control	Congestion and travel demand control with optimal time and DBC combined
Meng et al. (2012)	Any	DBC method of cordon-based congestion pricing (tested from S$0.5 to S$10/km for CBD cordon zone)	Total Social Benefits (TSBs) combining various operator and user benefits	Identify best parameter estimation range to link DBC toll charge with TSBs to determine related charges for policy making
Sen et al. (2021)	P	Testing factors decreasing commuting distance of Southeast Queensland to see if DBC is the best option to reduce commuting distance	Commuting distance predicted by travel related characteristics such as accessibility at residential and workplace, and individuals’ socio-economic characteristics No DBC amounts were tested	The findings conclude that DBC may not be the most effective method in reducing commuting distance, but accessibility of transport options at residential locations are
Yang et al. (2016)	Any	Test DBC alternatives based on fixed VMT, Ramsey pricing, fixed interval on income levels and fixed percentage on income levels were tested in Maryland US, from $1.10 to $7.76/mile tested	Maintain adequate fund to maintain transport system with equitable and progressive DBC	Variable DBC policies can achieve revenue goals. A fixed interval increase rate on people's income level is progressive overall

## Footnotes

1 These forecasts are based on pre-COVID-19 estimates of freight activity.

2 R-Tresis is in the development phase, but it uses the full SA3 by SA3 origin–destination network for all passenger modes (car, plane, rail and coach) and the key passenger demand models for three trip purposes (commuting, business and other (mainly tourism, personal business and visiting friend and relatives) for models representing modal share and the total number of trips by each mode which are linked using a logsum measure of accessibility. There is also a separate model to identify induced passenger travel demand.

3 Model systems that do not explicitly account for freight impacts typically reserve 20% of road capacity for freight movement.

4 Throughout Australia, bulk commodities tend to go by rail and non-bulk by road; although the former does use trucks for the first and last mile in many settings. https://www.bitre.gov.au/sites/default/files/is_034.pdf. Within an urban freight distribution setting, we do not expect a switch to rail; indeed we see no evidence that this will occur.

5 A referee suggested on average generally below 20% of distances driven and rarely above 50%.

6 The selection of Poisson or other models is judged by the data. Poisson is typically used with discrete count data but can be used to model a continuous variable if the variable is not negative and satisfies the Poisson requirement. The assumption an equal mean and variance is not so strict; just like regression can be used if the distribution is not exactly normal. If data is highly dispersed, then a negative binomial (NB) may be considered (Poisson is a special case of NB), but our model results predict well and we support the ZIP specification in modelling the kilo-tonne model. Adding an extra dispersion term of (log of) for distributed heterogeneity in the NB did not change the results.

7 1kt = 1000 tonnes.

8 We also tested for the residual from the empty truck share model and it was not statistically significant.

9 The ABS data is based on a sample survey of 16,000 articulated and rigid vehicles that were registered with an Australian motor vehicle registry during the collection period. Respondents were asked to provide information about their vehicle configuration, load carried and origin–destination for all trips undertaken within a randomly allocated reference week. See https://www.abs.gov.au/ausstats/abs@.nsf/mf/9223.0. SFM and TPA used ABS data and obtained unit record data to use in obtaining estimates of the variables not provided by ABS: https://opendata.transport.nsw.gov.au/dataset/freight-datahttps://data.datahub.freightaustralia.gov.au/it/dataset/strategic-freight-movement-model.

10 Statistical area level 3 (SA3) is one of the spatial units defined under the Australian Statistical Geography Standard (ASGS). This level of spatial details provides a very good strategic level representation of areas within metropolitan areas; for example the Greater Sydney Metropolitan Area has 80 zones, from the north in Newcastle to the south in Wollongong of which 46 are in the Sydney Area. The ASGS provides a common framework of statistical geography and thereby enables the production of statistics which are comparable and can be spatially integrated. There are 358 spatial SA3 regions covering the whole of Australia without gaps or overlaps (Appendix B). For details see. https://www.abs.gov.au/ausstats/abs@.nsf/Lookup/by%20Subject/1270.0.55.001~July%202016~Main%20Features~Statistical%20Area%20Level%203%20(SA3)~10015.

11 The kilo-tonne estimates are derived from the SFM and as such are forecasted estimates. We went to great pains to check the estimates based on a few available primary data on kilo-tonnes given that the primary input data was too sparse for all OD pairs.

12 The percentage of empty trips is often much higher, approaching 50%, in some urban areas in the US. We acknowledge this advice from Kouros (Abolfazl Mohammadian).

13 It is crucial that empty truck activity is represented by zero kilo-tonnes, otherwise there is a risk that the model will over-predict kilo-tonnes associated with truck movements in the other models associated with truck movements.

14 An appropriate assumption with a focus on predicting truck movements which in include many trucks not carrying commodities.

15 We are using very straightforward multinomial logit models, but with aggregated modal shares (or frequencies). The popular aggregate ‘choice’ model developed by Berry, Levinsohn and Pakes (BLP 1995) is not required here since users cannot influence the levels of the statistically significant explanatory variables, in aggregate. Several other papers have developed road vehicle type choice (or aggregate truck type share) models, such as Bok et al. (2020a, 2020b) and Abate et al. (2014).

16 We tested alternative specific parameters and they were not statistically different.

17 This is not an uncommon practice in toll road studies where a time equivalence is used for the toll.

18 We did estimate models assuming that kilo-tonnes was exogenous. Interestingly, the influence on all other parameter estimates in the aggregate truck type share model was negligible.

19 The assignment module in MetroScan utilises the open-source traffic assignment platform PLANit (https://github.sydney.edu.au/PLANit), developed at ITLS (University of Sydney). The assignment configuration is hard coded and conducts a traditional static traffic assignment with the following properties: Route choice and network loading is Deterministic User Equilibrium (DUE) following Beckman et al. (1956); the shortest path algorithm is Dijkstra one-to-all; smoothing uses the Method of Successive Averages (MSA); the number of iterations is user configurable; when set to 1 (default), DUE collapses to an All-Or-Nothing (AON) assignment; the duality gap threshold is user configurable; and the approach overall is based on Bovy and Jansen (1983).

20 For the application in Metroscan, we want to ensure that, for each OD pair and commodity-specific calculation, that the sign of the product of Logsum and its parameter estimate in the frequency model provides a directionally valid response to any change in one of more of the calculated estimates for Beta*Logsum. We found that the great majority of observations (i.e., OD pair by commodity class) result in the correct sign, and a negligible few OD pairs and commodity-specific observations had a different sign to that expected, largely associated with very low frequencies. Note also that the zero-inflated feature associated with Tau accommodates the probability of Poisson being non-zero versus the probability of zero occurring.

21 We have not discussed the Time of Day (ToD) component of the freight module in MetroScan. Currently, unlike the passenger modules, we do not have a ToD model and instead ‘allocate’ truck trips based on exogenously observed truck movements from traffic counts from an OD matrix in the GSMA.
